# Community health workers’ experiences of supervision by nurses at clinics in Limpopo Province

**DOI:** 10.4102/hsag.v28i0.2330

**Published:** 2023-12-08

**Authors:** Makwena M. Matsi, Eucebious S. Lekalakala-Mokgele, Mary M. Madumo

**Affiliations:** 1 Department of Nursing Sciences, Faculty of Health Care Sciences, Sefako Makgatho Health Sciences University, Pretoria, South Africa

**Keywords:** community health workers, experiences, nurses, primary healthcare facilities, supervision

## Abstract

**Background:**

Supervision of community health workers (CHWs) is considered, among others, a strategy to achieve universal healthcare globally. In South Africa, it is incorporated in the ward-based primary healthcare (PHC) outreach teams’ policy and strategy, a national health insurance policy component. Supervision of CHWs by nurses in the policy is considered a measure to facilitate PHC service provision to communities. However, CHWs experienced varying levels of supervision from nurses and other workers globally and in South Africa.

**Aim:**

This study explored and described the experiences of CHWs about supervision received from nurses at PHC facilities in Limpopo Province.

**Setting:**

Participants were drawn from seven PHC facilities in Polokwane and Lepelle-Nkumpi subdistricts of the Capricorn District.

**Methods:**

A qualitative exploratory-descriptive and contextual study design was employed. Participants were selected utilising a purposive sampling method. Semi-structured interviews were conducted to collect data. Data were analysed according to themes and their associated categories. Measures to ensure rigour and ethical principles were applied.

**Results:**

Two main themes emerged: positive supervision experienced by CHWs and supervision challenges experienced by CHWs.

**Conclusion:**

The varying experiences of CHWs about supervision from nurses emerged and reflected the need for functioning supervision mechanisms.

**Contribution:**

The experiences of CHWs indicated inconsistent delivery of supervision by nurses in PHC facilities. The findings highlighted the need for effective supervision measures that are vital for the success of the CHW supervision programme.

## Introduction

Community health workers (CHWs) play a vital role in the health system as they deliver primary healthcare (PHC) services close to communities. In South Africa, they perform this role under the supervision of nurses (outreach team leaders [OTLs]). Globally, supervision of CHWs has evolved over the last two decades (Crigler, Gergen & Perry 2013:2). The current emphasis is on the elimination of traditional supervision methods, which include inspection of performance, punishment of supervisees, irregular supervision and non-participation of supervisees in problem-solving (Marquez & Kean 2002:7).

A definition adopted in many studies indicates that efficient CHW supervision results in productivity through appropriate communication, problem-solving skills, teamwork and leadership (Adeyemo 2017:54; Assegaai & Schneider 2019:26; Marquez & Kean 2002:1; Westgate et al. 2021:15).

According to the World Health Organization (WHO), supervision of CHWs by health professionals is one of the measures to attain universal health coverage globally (WHO 2018:8). Likewise, in South Africa, supervision of CHWs by nurses is included in the ward-based PHC outreach teams’ policy and strategy. Community health workers are regarded as the first point of contact of the community to the health system (Kok et al. 2017:1). Hence, the provision of quality supervision by nurses is a tool to fast-track PHC services delivery in the households and thus the attainment of the National Department of Health’s (NDoH’s) policy (NDoH 2018:3).

Studies reported that CHW supervision is not appropriately executed (Assegaai & Schneider 2019:3; Hill et al. 2014:2). The inconsistencies in its application occur amid the availability of guidelines for its effective implementation (ed. Cutcliffe 2015; Hodgins, Crigler & Perry 2018:7; WHO 2018:45). Similarly, in South Africa, supervision of CHWs by nurses has been reported to be implemented poorly in the ward-based PHC outreach teams’ policy due to a lack of details or guidelines, as well as the need to improve on it (Assegaai & Schneider 2019:31; Munshi et al. 2019:7; NDoH 2018:18).

The CHWs reported different experiences of supervision in research studies, mostly in low- and middle-income countries (LMICs). Supervision encounters during field visits involved activities like performance assessment and exposure to useful feedback strategies (Roberton et al. 2015:24; Tseng et al. 2019:6). Addressing household challenges was also stated as part of supervision (Assegaai & Schneider 2022:36). Experiences of decent working relationships with facility staff instigated by the nurse supervisors were also reported. The outcomes of supervision, such as enhanced communication and productivity, were also uttered by the CHWs (Tseng et al. 2019:7). Other experiences of CHWs included expressions of supervision encounters in meetings with supervisors. The supervisory dealings in the meetings included reports checking, feedback sessions and capacity building (Roberton et al. 2015:24). Several authors also indicated that the CHWs experienced rigorous monitoring of working hours through team supervision (Mottiar & Lodge 2018:56; Ndima et al. 2015:69). The findings from the studies mentioned earlier showed the application of some aspects of the definition of supervision concerning communication, problem-solving skills, teamwork as well as leadership through individual and group supervision.

Nevertheless, research studies also reported undesirable experiences of CHW supervision by nurses. Some CHWs indicated that supervision visits were non-productive due to the supervisor’s lack of effective problem-solving skills, and this ultimately affected their productivity (Roberton et al. 2015:24; Tseng et al. 2019:6). Moreover, some CHWs were reportedly deprived of supervision due to attrition of supervisors, unplanned visits and policing supervisory practices (Kok et al. 2018:996; Silva et al. 2014:897). Similarly, in South Africa, CHWs reported that the absence of supervision by nurses limited their ability to provide PHC services in communities due to inadequate training and the ensuing demotivation (Motswasele-Sikwane et al. 2020:4; Tseng et al. 2019:6 & 7).

In another South African study, CHWs felt unwelcomed by facility staff as depicted by poor communication and a lack of working space and thus indicative of inadequate supervision (Munshi et al. 2019:4). A study also indicated CHWs’ expressions of the absence of supervision and offence by their superiors; hence, strengthening supervisor-supervisee training to mend working relationships was proposed (Kok et al. 2017:5). These results highlighted the experiences of inadequate delivery of supervision and the resulting impact on CHWs as well as on PHC services delivery.

The experiences of CHWs show some discrepancies in the appropriate delivery of supervision and the resulting impact on the overall programme outcomes. These may negatively impact major projects such as universal health coverage and national health insurance, from which the CHW supervision programme emanates. Endeavouring to close this gap, the researchers explored and described the experiences of CHWs about supervision by nurses in the Capricorn District, Limpopo Province. The findings reported in this article provide a foundation for the second phase of the main study: the development of a CHW supportive supervision programme for nurses. This article, therefore, addressed the following study question: what are the experiences of CHWs about supervision provided by their nurse supervisors?

## Problem statement

The contribution of the CHW programme towards achieving health outcomes is reported globally, such as in the South Africa’s policy framework for ward-based PHC outreach teams and the WHO CHWs guidelines. The attainment of such outcomes is attributed to factors such as effective CHW supervision. However, studies of different magnitudes from LMIC countries, including South Africa, indicated the disparities in the execution of CHW supervision with samples comprising of CHWs (*n* = 37–2155) as well as the resulting impact on the attainment of universal health coverage (Assegaai & Schneider 2019:28; Ludwick et al. 2018:13; Munshi et al. 2019:3; Tseng et al. 2019:2). The inconsistent supervision of CHWs is also highlighted in Limpopo Province, a rural province with mostly poor communities (Mashele 2021:75; Murphy et al. 2021:5). The researcher has also noted from discussions with nurse supervisors, CHWs and colleagues that the supervision of CHWs is not implemented consistently, especially in line with the recommendations stipulated in the ward-based PHC outreach teams’ policy and WHO CHWs’ training modules and guidelines, respectively (National Department of Health [NDoH] 2018: World Health Organisation [WHO] 2020).

Based on the identified gaps, the researcher explored and described the experiences of CHWs about supervision by nurses in the Capricorn District, Limpopo Province. These experiences might assist in providing effective supervision to CHWs, eventually attaining CHW supervision programme goals and major projects such as national health insurance and universal health coverage.

## Research methods and design

### Study design or approach

A qualitative exploratory-descriptive and contextual study approach was employed. The application of the design addressed one of the objectives of the main study, namely, to explore and describe the experiences of CHWs regarding the supervision provided by nurses at PHC facilities in Limpopo Province (Gray, Grove & Sutherland 2017). The naturalistic paradigm formed the basis of this inquiry. The inquirer examined subjective and complex human experiences in the context and natural setting in which they occur (McInnes et al. 2017). The researcher was concerned with how the subjects constructed various realities and attempted to reconcile those realities (Moschkovich 2019:61). Data were collected from CHWs posted in different villages and attached to numerous PHC facilities (Table 1). Thematic data analysis was used to understand the participants’ experiences. The findings reported in this article will be incorporated into the CHWs’ supportive supervision programme for nurses, which is the ultimate purpose of the main study.

**TABLE 1:** Distribution of community health workers sampled in primary healthcare.

Subdistrict	PHC facility number	Number of CHWs
Polokwane	1	3
	2	3
	3	3
	4	3
	5	2
**Subtotal**	**-**	**14**
Lepelle-Nkumpi	6	4
	7	3
**Subtotal**	**-**	**7**
**Grand total**	**-**	**21**

PHC, primary healthcare; CHW, community health workers.

### Setting

The study was conducted at clinics in two of the four subdistricts of Capricorn District, Limpopo Province, namely, Polokwane and Lepelle-Nkumpi. Participants were drawn from five clinics at the Polokwane subdistrict, and at the Lepelle-Nkumpi subdistrict, two clinics were sampled. Only PHC clinics with CHWs conducting fieldwork were included in this study. The participants from these facilities were relevant to inform the study about the supervisory responsibilities of the nurse supervisors or OTLs. Healthcare centres and clinics with CHWs not conducting fieldwork were excluded in this study. The interviews were conducted at the nurses’ residence, manager’s office, consultation rooms or kitchen in PHC facilities. The participants were primarily drawn from the rural clinics because the subdistricts’ PHC facilities were predominantly located in those areas.

### Study population and sampling strategy

#### Population

The population comprised all the CHWs in the Capricorn Health District. From this population, CHWs who worked for at least 1 year at the clinics were sampled purposively. The estimated number of CHWs was 1535 as of April 2021 according to the Capricorn District quarterly CHWs database (DoH 2021). Further division of the CHW population per subdistrict was as follows: Polokwane (*n* = 715); Molemole (*n* = 171); Blouberg (*n* = 273) and Lepelle-Nkumpi (*n* = 376).

#### Sampling

A lack of practical guidance about determining sample sizes in qualitative studies has been reported previously (Guetterman 2015:2). The preliminary sample size was 30; 21 participants were purposively sampled, and this was informed by data saturation (Brink, Van Der Walt & Van Rensburg 2018:141). Only CHWs with work experience of a year or more, supervised by nurses at designated clinics and performing fieldwork in communities, were included in this study. Community health workers with less than a year’s work experience were excluded in this study. Moreover, CHWs employed by non-governmental organisations and those not performing fieldwork in communities were also excluded.

### Data collection

The researcher recruited participants with experience of nurse supervision through the assistance of the managers of PHC facilities. The managers provided the researcher with a list of the prospective study participants who were recruited individually by the researcher. The researcher introduced herself in person to the prospective participants, verbally informed them about the study and issued study information leaflets written in the language (Sepedi) they understood. Upon receiving study information, all participants willing to participate were requested to provide written informed consent. Face-to-face interviews were conducted by the student researcher from February 2022 to July 2022. Participants were interviewed at the PHC facilities (nurses’ residence in the healthcare facility, manager’s office, consultation rooms or kitchen) on dates agreed upon with the facility managers and participants. Participants were interviewed during working hours on the days they were allocated to work in clinics as per prior arrangements with the facility managers. Interviews ranged from 30 min to an hour. Data were collected using a semi-structured interview guide that allowed the researcher to explore the participants’ thoughts, feelings and beliefs about supervision received from nurses (DeJonckheere & Vaughn 2019). The guide was written in Sepedi and English, the most used languages in the study setting. All participants opted to be interviewed in the Sepedi language to express their experiences best. Sepedi was also the researcher’s native language, and this assisted in collecting in-depth data from the participants. An audio recorder, a cell phone and a memo pad were used to record the interviews. Listening, probing, reflecting, clarifying and paraphrasing skills were employed to collect in-depth participant data. Data were transcribed verbatim and translated into the English language by the student researcher. Moreover, during the data collection process, coronavirus disease 2019 (COVID-19) protocols were adhered to. The participants responded to the following questions during the interviews as per this guide:

- **Central question:**
  ■ What are your experiences of supervision provided by your nurse supervisor?
- **Probes:**
  ■ Which supervision activities are provided to you by the nurse supervisors as the CHW?
  ■ What challenges are you experiencing regarding the supervision you receive from the nurse supervisor?

### Data analysis

The researcher transcribed participants’ data in audio and memo pad recordings verbatim (Sepedi) and then translated it into English language. Data were back translated into Sepedi language by the researcher because she was bilingual to ensure that the translation was correct. Tesch’s qualitative data analysis method was utilised to identify themes and their related categories. The researcher familiarised herself with the data by reading the transcripts more than once and identifying codes (Brink et al. 2018:193). Data were independently analysed by the co-coder for identification of themes, categories and subcategories. This was followed by a consensus meeting between the co-coder and researcher and then finalising study findings (Grodal, Anteby & Holm 2021; Harding & Whitehead 2013:137). The researcher also engaged in an intellectual exercise to determine whether the identified themes answered the research question.

### Ethical considerations

The researcher obtained approval from the Sefako Makgatho Health Sciences University Ethics Committee before data collection (Reference: SMUREC/H/144/2021:PG). Permission to collect data from the clinics was granted by the Limpopo Province Department of Health Research Ethics Committee and the Capricorn Health District Management. The PHC facilities’ operational nursing managers (OPMs) allowed the researcher to gain access to participants upon presentation of the permission letters. The research adhered to the ethical principles of openness and integrity, beneficence, confidentiality and privacy, and justice during the study. The study’s nature and breadth were explained to the participants in Sepedi, as per the participants’ preference. Moreover, the participants were informed of their freedom to participate voluntarily or withdraw anytime from the study. Upon explanation of the entire study, written informed consent was obtained from the participants before data collection. Participants were informed that they do not have to answer any question they are uncomfortable with. In cases of questions that provoke emotional distress, the researcher told participants that the interview would be suspended and they would have the opportunity to be debriefed. Participants were also informed that the suspended interview may be rescheduled for a later date.

### Trustworthiness

The researcher applied the criteria of credibility, transferability, confirmability, authenticity and dependability to ensure the study’s trustworthiness. The researcher applied credibility by interviewing each participant for at least 30–60 min according to the developed interview guide and focusing on data saturation. Data were collected through interviews with CHWs at different clinics and subdistricts using an audio recorder, cellphone and memo pad. The data of the entire study were validated with the supervisors. To ensure transferability, the researcher provided detailed information about the study setting, participants and procedure of data collection. Readers of this study may apply them to their own context. Data were collected from CHWs of different age groups and gender (only one male participated in this study), but there was no significant difference. In achieving confirmability, the researcher reflected only on the participants’ responses, maintained an audit trail of the data collection and analysis process and applied reflexivity by shelving any preconceived thoughts about the entire study. The researchers had no previous relationships with the participants before the data collection process. Data triangulation was achieved by collecting data from participants working in different clinics at the three subdistricts of the Capricorn District, recording interview data on an audio recorder, cellphone and memo pad and ensuring that data were analysed by the researcher and co-coder. The researcher also reported the data correctly as narrated by the participants; hence, some of the participants’ quotations are reflected in this article to show authenticity. Dependability was enhanced by using quality audio recorders and taking field notes of the participants’ non-verbal cues during the interviews.

### Bias

Bracketing was addressed by noting participants’ data in a memo pad and shelving cognitive and affective preconceptions. Moreover, the researcher deferred any beliefs which could have negatively impacted the study. Reflexivity was achieved through self-introspection and reflection on possible personal and contextual biases that may have affected data collection, analysis and interpretation. Participants’ response bias was dealt with by explaining the purpose and significance of the study as well as clarifying relationships during data collection. Reporting bias was avoided by not generalising the findings because the participants were sampled through a non-random purposive sampling technique.

## Results

### Participants’ biographic summary

Twenty-one participants were interviewed – 14 were from the Polokwane subdistrict and 7 from the Lepelle-Nkumpi subdistrict. Among the 21 participants, the majority were female (*n* = 20). Most participants were in the age category below 40 years (12 out of 21), followed by 40–49 years (5 out of 21) and 50 years and above (4 out of 21). Fourteen participants had grade 12 certificates, indicating that most of the participants accomplished basic schooling. All participants reported receiving basic CHWs training. Although some participants said they were contract employees, most perceived their employment status as temporary. All participants worked for more than a year in the designated clinics.

### Themes, categories and subcategories

Participants’ responses of supervision experiences from nurses were arranged into themes, categories and subcategories (Table 2).

**TABLE 2:** Themes, categories and subcategories of community health workers’ experiences of supervision provided by nurses.

Themes	Categories	Subcategories
1. Positive supervision experienced by community health workers	1.1. Supervision of community health workers	1.1.1. Access and supervision during fieldwork 1.1.2. Supervision with complicated or resistant cases 1.1.3. Supervision with work relationships 1.1.4. Supervision discussions and meetings 1.1.5. Monitoring of daily, weekly and monthly reports 1.1.6. Capacitation of community health workers
2. Supervision challenges experienced by community health workers	2.1. Challenges related to community health worker supervision	2.1.1. Inadequate supervision due to supervisor’s inaccessibility 2.1.2. Inconducive approach to supervision 2.1.3. Inappropriate utilisation of community health workers 2.1.4. The consequences of inappropriate delegation of community health workers 2.1.5. Ineffective communication strategies

#### Theme 1: Positive supervision experienced by community health workers

The participants’ data resulted in this theme. One category was identified from the participants’ descriptions, with six related subcategories.

**Category 1.1: Supervision of community health workers:** The participants narrated experiences of their supervision by the nurses. They mentioned that the supervisors were accessible to provide supervision during fieldwork in communities. The participants’ statements further showed that their experiences of supervision from nurses encompassed supervision with complicated or resistant cases, supervision with work relationships, supervision discussions and meetings, monitoring of daily, weekly and monthly reports and capacitation of CHWs.

***Subcategory 1.1.1: Access and supervision during fieldwork:*** The supervision experiences of participants pointed to the accessibility of nurses while providing PHC services to the communities. During the supervisory visits, the participants said the nurses evaluated their performance, assessed the cases they referred and provided health education in the households and during door-to-door campaigns.

Participants’ utterances about access and supervision during fieldwork were as follows:

‘The nurse supervisor checks how I greet the people, whether I’m maintaining eye contact or I talk with people while using the phone, whether I’m completing the forms correctly and how I ask questions. The nurse supervisor will be assessing me using a checklist with 8 components and in case I left something out the nurse supervisor will tell me when we are out of the household.’ (P9, 42 yrs. old, CHW trained)

‘We agree on the day, depending on what I have observed and reported. She will then come with either me or the two of us to supervise us where she also provides support to the family on what to do in relation to something. Truly, she does support them.’ (P21, 49 yrs. old, CHW trained)

***Subcategory 1.1.2: Supervision with complicated or resistant cases:*** Participants also voiced the provision of supervision with challenging patients or family-related cases during supervisory visits. In addition, they indicated that the nurses provided advice, handled the cases personally or referred client(s) to other health professionals or levels of healthcare. Some participants also stated that the nurses prioritised the clients they referred to the clinic and later communicated the care rendered.

The participants’ statements were as follows:

‘The nurse supervisor is very good when it comes to referral. She often attends to our referrals very quickly when she notices that it is from me, and then she informs me when she has attended to the people we referred.’ (P8, 38 yrs. old, CHW trained)

‘When I encounter challenges at the field. Sometimes, she assists me there and then by guiding me on what I must do, or she can also help me by handling the challenge.’ (P20, 45 yrs. old, CHW trained)

***Subcategory 1.1.3: Supervision with work relationships:*** The participants indicated that the nurses tackled strained work relationships among the CHWs or between the CHWs and facility staff. Furthermore, the participants narrated that the nurses resolved those tensions individually or with the clinic’s operational nurse managers. Strategies that involved the parties concerned only or engaging with all the facility staff were also highlighted.

The participants said:

‘The outreach team leader and operational manager addressed all the sisters that they are the ones who are in the wrong and said when they are not at work, we still needed the in-service training. They said that they must call an individual community health worker and show him or her how to fill in those forms.’ (P8, 38 yrs. old, CHW trained)

‘When it comes to work-related issues, the nurse supervisor can call all parties involved and address the issue that we face respectfully. The nurse supervisor once addressed issues like gossip where one person was hurt by what the other colleague had said, and then she advised that we are a family and we must think about others’ feelings with whatever we say.’ (P11, 55 yrs. old, CHW trained)

***Subcategory 1.1.4: Supervision discussions and meetings:*** The participants stated that their supervision encounters included meeting with the nurse supervisors. Once more, they said the focus was mainly on resolving fieldwork-related challenges. However, one participant reported that they met in the absence of the nurse supervisor and proposed ways to resolve the challenges they experienced.

Participants’ quotations are as follows:

‘When we come from there [*field*], as you have heard that, on Fridays we are not going to the field. We give her the challenges we had during the week saying “We had problems like this one and that one” that is the support we receive for now.’ (P1, 49 yrs. old, CHW trained)

‘Yes, on a monthly basis, we can meet with the nurse supervisor to present all the challenges we experienced from various areas we work so that she can solve them as our outreach team leader.’ (P13, 57 yrs. old, CHW trained)

***Subcategory 1.1.5: Monitoring of daily, weekly and monthly reports:*** Regarding work reports, the participants reported that the nurses mainly checked the fieldwork to determine and validate work attendance. One participant further indicated that report validation was essential to ensure the correctness of the written data.

The participants uttered the following:

‘Our supervisor, when we will be compiling our weekly reports, she will be checking the work that we did from Monday to Thursday. The nurse supervisor will be checking whether I went to the household so and so.’ (P4, 46 yrs. old, CHW trained)

‘The supervisor wants to see if the numbers are balancing. Isn’t sometimes I can make wrong entries? I can write that I have seen a child under the age of one, but on the other side, I write zero.’ (P6, 34 yrs. old, CHW trained)

***Subcategory 1.1.6: Capacitation of community health workers:*** According to the participants, the nurse supervisors capacitated them to improve their knowledge and skills. They also mentioned that they applied strategies such as in-service education, on-spot teaching and health talks.

Participants’ expressions were as follows:

‘The support we receive from the outreach team leader is that she provides in-service training also with our reports, where we don’t understand something in our reports, we go to her so that she can explain better that when something is like this, we do this. She does the in-service training now and then. Today, we were also supposed to do in-service training on something related to our work. Isn’t there are a change now and then, so there are some that we were never in-serviced on before.’ (P14, 44 yrs. old, CHW trained)

‘Where we don’t understand, the nurse supervisor assists us, especially with certain things we are not trained for and not clear to us. The nurse supervisor does guide us on how it’s done.’ (P19, 45 yrs. old, CHW trained)

#### Theme 2: Challenges related to supervision

This theme resulted from the participants’ descriptions. One category emerged from this theme, and five associated subcategories.

**Category 2.1: Challenges related to community health worker supervision:** The participants described challenges relating to the provision of supervision by the nurses. It was apparent that the nurse supervisors were sometimes inaccessible to assist with fieldwork challenges. Other challenges included the supervisors’ lack of knowledge, inability to make decisions, use of unfavourable supervision approaches and delegated duties outside the scope of work. The associated consequences were also articulated.

***Subcategory 2.1.1: Inadequate supervision due to organisational challenges:*** The participants’ expressions highlighted that the nurses were unreachable when needed in the field, mainly due to a lack of transport and prioritisation of clinic duties.

Participants’ quotations are as follows:

‘Our supervisor supports us in a good way because when we find challenges we report to her. The problem is that there is a lot of work and staff shortage in the clinic, so she is unable to come with us to the field.’ (P3, 45 yrs. old, CHW trained)

‘Since she left [*supervisor from ANOVA* {*non-governmental organisation*}], our supervisor never came with us to provide supervision. I don’t understand why, but I have heard that he has a challenge with transport; hence, he doesn’t come to us.’ (P5, 37 yrs. old, CHW trained)

***Subcategory 2.1.2: Inconducive approach to supervision:*** The participants’ utterances showed that there was poor communication by the nurse supervisors, indicated by attitudes of being disrespectful and intimidating. Some of the supervisors were regarded as unapproachable, resulting in the participants being unable to seek clarity regarding questionable work practices. Others indicated that the nurses failed to appreciate their work and, as such, felt demotivated.

The participants said:

‘It’s just that he [*nurse supervisor*] is the kind of a person when you make mistakes … according to me, if a person makes mistakes, it’s not necessary to shout her, you must sit down with the person and correct her, but he sometimes shouts and when he does that the others [*community health workers*] laugh.’ (P6, 34 yrs. old, CHW trained)

‘Yes, the nurse supervisor may come, but we [*community health workers*] feel like she is checking if we didn’t go off. Like when we work, we don’t feel like she supports us because she never appreciate our work, like saying, “Today we really worked, today we did something good.” We have never heard something like that, especially when we work at the clinic; they only see us when we make mistakes.’ (P16, 38 yrs. old, CHW trained)

***Subcategory 2.1.3: Inappropriate utilisation of community health workers:*** The participants stated that they were forced to perform tasks at the clinic which were out of their scope of work. They performed the tasks in case of a shortage of clinic personnel and felt that it was inappropriate because they were hired to do fieldwork.

The participants’ quotations follow:

‘I’m not fine with it because the money I get here is not even enough for me to measure BPs [*blood pressures*] here. It [*the money*] is not enough for me to work here [*at the clinic*], but to work with patients at the field.’ (P2, 42 yrs. old, CHW trained)

‘Sometimes, they [*nurse supervisors*] make us remove weed from the ground. We asked ourselves why because there are groundsmen in here, we are afraid of asking them. But we end up not having the answer.’ (P5, 37 yrs. old, CHW trained)

***Subcategory 2.1.4: The consequences of inappropriate delegation of community health workers:*** The participants mainly indicated that being delegated to perform non-CHWs duties at clinics hindered them from providing PHC services to the communities, negatively impacting their productivity and morale.

Participants’ quotations are given below:

‘So, if we spend more days here with only one day for fieldwork, we end up having more work to do. This causes us to have stress because our statistics are low and the outreach team leader is also complaining.’ (P1, 49 yrs. old, CHW trained)

‘We don’t feel good because when I come to work, sometimes when I’m supposed to go to the field, the nurses say they are short-staffed. I must assist while my fieldwork is halted. When the week ends, they need a report; where will I get it because I’m with them [*nurses*] here [*at the clinic*].’ (P20, 45 yrs. old, CHW trained)

***Subcategory 2.1.5: Ineffective communication strategies:*** The participants’ narratives revealed that the nurse supervisors were not communicating clearly with them. They said some nurse supervisors failed to address the challenges related to their work and attendance at labour relations activities. Consequently, they felt helpless, demotivated and then engaged in inappropriate conduct.

Excerpts from the participants’ narrations follow:

‘Sometimes, we end up not reporting that “like tomorrow we are going for a march” because at times the nurse supervisors won’t allow us. So those who work at the clinic will remain working; those who are at the field [*on the day of the march*] we go.’ (P7, 47 yrs. old, CHW trained)

‘But if I report but I’m not heard, I can say that I’m not receiving support and become demoralised.’ (P13, 57 yrs. old, CHW trained)

## Discussion

According to the WHO, effective supervision is vital to improving the performance of the CHWs supervision programmes. It is also welcomed by CHWs (WHO 2018:48). Supervision of CHWs by nurses in South Africa is included in the ward-based PHC outreach teams’ policy and strategy, a component of the PHC reengineering strategy of the national health insurance policy (NDoH 2018:3 & 5). Within the ward-based PHC outreach teams’ policy, supervision of CHWs is intended to enhance PHC service delivery to communities (NDoH 2018:17 & 18). However, CHW supervision is regarded to be ineffectively implemented globally (Assegaai & Schneider 2019:3; Hill et al. 2014:2).

In the current study, CHWs’ experiences of supervision were inconsistent. Positive supervision experiences included CHWs’ utterances relating to nurse supervisor access during fieldwork, assistance with challenging community cases, tackling strained work relationships, engagement in supervision meetings, capacitation, monitoring of work reports and compliance with work standards. Negative sentiments were also expressed by the CHWs related to supervisors’ inaccessibility and poorly trained and disempowered nurse supervisors. Use of inconducive approaches to supervision, inept delegation and the consequences of supervision challenges also emerged.

The scope of work and job description of the OTLs or CHWs’ nurse supervisors stated that every month, they ought to spend 70% of their time conducting supervised household visits with the CHWs (NDoH n.d:2). It further indicated that the remaining 30% is meant for the administrative aspect of supervision of the CHWs (NDoH n.d:2). This clearly showed that the nurse supervisors are expected to spend most of their work time in ensuring that the CHWs perform their duties in communities. Box 1 outlines the supervisory roles and responsibilities of the nurses of OTLs and the duties of CHWs (Murphy et al. 2021:385; NDoH n.d).

BOX 1 The scope of supervision of the nurse supervisors and duties of the community health workers.

**Scope of supervision of nurse supervisors**

Plan daily and weekly activities, including home visits with the community health workers.

Conduct regular and irregular household visits to ensure continuous supervision of community health workers.

Assess community health workers’ understanding of the content and engagement with households during visits.

Identify problem cases in the households, develop an improvement plan with the community health workers, and facilitate linkage to care.

Monitor the implementation of the improvement plan by the community health workers.

Identify community platforms and meetings to inform the community of community health workers’ activities.

Conduct or support community interventions and campaigns conducted by the community health workers.

Ensure that the community health workers have the tools to carry out their daily activities and the safekeeping thereof.

Compile weekly and monthly reports of the community health workers’ job performance.

Ensure that the community health workers report for duty.

Keep the duty and leave register for the community health workers.

Monitor community health workers’ performance according to their performance management requirements and report on progress.

**Duties of community health workers**

Conduct community, household and individual health assessments and identify health needs and risks.

Facilitate the family or an individual to seek the appropriate health service.

Promote the health of the households and the individuals within these households. Refer persons for further assessment and testing after performing simple primary screening.

Provide limited, simple health interventions in a household.

Provide psychosocial support.

Manage interventions such as treatment defaulter tracing and adherence support.

*Sources:* Murphy, J.P., Moolla, A., Kgowedi, S., Mongwenyana, C., Mngadi, S., Ngcobo, N. et al., 2021, ‘Community health worker models in South Africa: A qualitative study on policy implementation of the 2018/19 revised framework’, *Health Policy and Planning* 36(4), 384–396. https://doi.org/10.1093/heapol/czaa172; National Department of Health, n.d., *Job description and scope of work ward based primary healthcare outreach team leader*, National Department of Health, Pretoria

In this study, the CHW expressions showed that the nurses were available to assist with patients and family challenges, conducted door-to-door campaigns and provided health education in communities. Consistent findings were reported in studies where the CHWs reported experiences of exceptional work bonds with the nurse supervisors during the household visits (Assegaai & Schneider 2019:31; Roberton et al. 2015:25; Tseng et al. 2019:6). Remarkably, the presence of the nurse supervisors was perceived as a mechanism that strengthened CHWs–community relations, hence the acceptability of the CHW supervision programme (Assegaai & Schneider 2019:31; Ndima et al. 2015:68). Moreover, the nurses also acknowledged their supervision role to assist the CHWs with household challenges (Assegaai & Schneider 2019:31; Malatji et al. 2022:330).

The provision of health education to clients during field visits and engaging in door-to-door campaigns by the CHWs’ supervisors were reported in studies (Mhlongo & Lutge 2021:1723; Roberton et al. 2015:25). Nevertheless, some CHWs in this study said the visits only occurred when they report household challenges. A lack of transport, nursing staff shortage and uncommitted nurse supervisors were mentioned as supervision-hindering factors. These results echo findings in studies where the CHWs experienced irregular supervision of fieldwork due to supervisors performing numerous responsibilities in the face of logistical challenges such as the lack of transport (Lister et al. 2017:5; Ndima et al. 2015:69; Roberton et al. 2015:23). Consequently, the CHW supervision programme was impacted (Motswasele-Sikwane et al. 2020:4; Ndima et al. 2015:69). The results in this study indicate a supervisory challenge that hinders the clients’ linkage to care (Limpopo Department of Health n.d:2).

Regarding relationships with staff at PHC facilities, the CHWs stated that the nurses resolved issues involving work-related tensions individually or with the facility managers. Poor relationships between CHWs and facility staff were widely reported in studies (Lister et al. 2017:5; Malatji et al. 2022:330). Malatji et al. (2022:330) reported that the nurses addressed the conflict between the CHWs and nurses emanating from patient tracing by regulating the number of CHW household visits, and this resulted in improved relations. In another study, Mhlongo and Lutge (2021:1723) mentioned that the nurse supervisor’s attempts to resolve conflict remained futile, which contradicts the findings in this study.

The CHWs are to report their task achievements, problem areas and fieldwork supervision outcomes during meetings with nurses (Mhlongo & Lutge 2021:1723). The descriptions of the CHWs disclosed that fieldwork challenges were discussed and the related resolutions made. These findings confirmed the execution of an aspect of the nurse supervisors’ scope of work. The nurses again checked fieldwork records weekly and monthly to verify the achievement of work responsibilities described by the CHWs. Studies conducted in Mozambique and South Africa revealed more or less the same results in which a team of supervisors (facility and community or non-governmental-based) checked the reports to evaluate CHWs’ work attendance and accomplishment (Mottiar & Lodge 2018:56; Ndima et al. 2015:69). The CHWs also verbalised that checking of their work reports boosted their report writing skills and were also clarified on their work records (Roberton et al. 2015:25).

In the current study, the CHWs’ responses showed that the nurse supervisors implemented in-service education, on-spot teaching and health talks to capacitate them. Compatible results from research in African countries mentioned that CHWs experienced supervision through continuous education and training coupled with mentorship. These resulted in CHWs acquiring new knowledge and motivation (Kok et al. 2018:995). These results coincide with an aspect of the scope of work of nurse supervisors, that of supporting CHW training (LDoH n.d:2).

Challenges related to the supervision of CHWs are also discussed in several studies (Assegaai & Schneider 2019:26; Avortri et al. 2019:5; Roberton et al. 2015:19). In the current study, the CHWs expressed concerns over the supervisor’s knowledge deficit which compromised decision-making and command ability. Thus, a study in LMIC suggested the need for the capacitation of supervisors and joint decision-making as requisites for efficient CHW supervision (Avortri et al. 2019:3). This proposal might also be beneficial to some CHWs and their nurse supervisors in this study.

The use of inappropriate approaches to supervision, such as poor communication and performance monitoring, was also uttered by the CHWs. Other authors specified analogous results and further showed that the CHWs felt unsupported and affronted by their superiors, as well as the negative impact on their performance and motivation. Therefore, combined supervisor-supervisee training focused on role clarification was recommended (Kok et al. 2017:5).

The CHWs voiced dissatisfaction with delegation of duties outside their scope of work by the nurse supervisors and its harmful effects on their overall work outcomes. In a Malawian inquiry, CHWs reported encounters with a broad scope of work due to the performance of additional tasks and the associated poor productivity due to work stress (Ndambo et al. 2022:185). In addition, the CHWs in recent South African studies felt unwelcomed by some staff members as they were perceived as threats to their work and affronted (Assegaai & Schneider 2022:35; Murphy et al. 2021:393).

### Strengths and limitations

The methodology allowed the participants to provide data within their work context. As such, they were best positioned to share their supervision experiences in their natural setting. However, the experiences of the CHWs cannot be generalised because the study was contextual. The other weakness is that mostly females participated in this study; if more male CHWs were interviewed, they may have reported different experiences. The researcher also collected data from a few participants. Data were collected in the clinic setup, and the setting might have compromised the freedom of expression of some participants.

## Recommendations

The participants’ experiences indicated interesting and variable findings. Although there are positive supervision practices which must be sustained, the CHWs also expressed statements that require responsiveness through the application of appropriate supervision strategies. This is important, especially with the CHWs who are regarded as a link of the health systems to communities for PHC services delivery globally and in other national programmes. Hence, it is imperative that supervision of CHWs by nurses be prioritised. An inclusive programme considering the human and non-human factors mentioned in this study could contribute towards a successful CHW supervision programme.

## Conclusion

This study pointed to the inadequacies in CHW supervision by nurses and the hindering factors. The findings contradict the standards for effective CHW supervision highlighted in the OTL scope of work and job descriptions and in the ward-based PHC outreach teams’ policy and strategy, among others. The inadequacies in supervision weaken the effective delivery of PHC service to communities by the CHWs and nurse supervisors. Hence, effective CHW supervision by nurses must be prioritised as a requisite for the successful CHW supervision programme and, consequently the national health insurance in South Africa. The realisation of this goal necessitates that the requisite human and non-human means are consistently available, central for its appropriate implementation and attainment of the desired outcomes.
